# Elucidation of the Molecular Mechanism Underlying *Lippia citriodora*(Lim.)-Induced Relaxation and Anti-Depression

**DOI:** 10.3390/ijms20143556

**Published:** 2019-07-20

**Authors:** Mouad Sabti, Kazunori Sasaki, Chemseddoha Gadhi, Hiroko Isoda

**Affiliations:** 1 Alliance for Research on the Mediterranean and North Africa (ARENA), University of Tsukuba, 1-1-1 Tennodai, Tsukuba City 305-8572, Ibaraki, Japan; 2 Tsukuba Life Science Innovation Program (T-LSI), University of Tsukuba, Tennodai 1-1-1, Tsukuba City 305-8577, Ibaraki, Japan; 3 Interdisciplinary Research Center for Catalytic Chemistry, National Institute of Advanced Industrial Science and Technology (AIST), Tsukuba 305-8560, Japan; 4 Faculty of Sciences Semlalia, Cadi Ayyad University, Avenue Prince MoulayAbdellah, BP 2390, 40000 Marrakesh, Morocco

**Keywords:** *Lippia citriodora*, VEE, Vs, relaxation, depression, mitochondria, cyclic AMP, calcium

## Abstract

*Lippia citriodora* ethanolic extract (VEE) and verbascoside (Vs), a phenypropanoid glycoside, have been demonstrated to exert relaxant and anxiolytic properties. However, the molecular mechanisms behind their effects are still unclear. In this work, we studied the effects and action mechanisms of VEE and Vs *in vivo* and *in vitro*, on human neurotypic SH-SY5Y cells.TST was conducted on mice treated orally with VEE (25, 50 and 100 mg/Kg), Vs (2.5 and 5 mg/Kg), Bupropion (20 mg/Kg) and Milli-Q water. Higher dose of VEE-treated mice showed an increase of immobility time compared to control groups, indicating an induction of relaxation. This effect was found to be induced by regulation of genes playing key roles in calcium homeostasis (calcium channels), cyclic AMP (cAMP) production and energy metabolism. On the other hand, low doses of VEE and Vs showed an antidepressant-like effect and was confirmed by serotonin, noradrenalin, dopamine and BDNF expressions. Finally, VEE and Vsenhancedcell viability, mitochondrial activity and calcium uptake *in vitro* confirming *in vivo* findings. Our results showed induction of relaxation and antidepressant-like effects depending on the administered dose of VEE and Vs, through modulation of cAMP and calcium.

## 1. Introduction

The Verbenaceae, commonly known as the verbena or vervain family, is composed of 35 genera containing around 1200 species [1]. They have been used for centuries as medicinal plants due to their beneficial effects to cure several ailments. One of the most important genera is *Lippia*, consisting of200 species exerting interesting biological activities [2]. *Lippia citriodora* K., also referred to as *Aloysiatriphylla*(L’Herit.), is commonly named lemon verbena, vervain or Louisa (Arabic). This species is native to South America and has been cultivated in Europe and North Africa mainly in Morocco [3]. All over Morocco, the plant is used as relaxant and sedative [4]. The herbal tea is traditionally used to alleviate insomnia and restlessness in adults as well as babies [5]. Furthermore, it has been used for its anti-inflammatory, antioxidant, antispasmodic effects and also used as a remedy for gastrointestinal disorders [2]. Recent studies have confirmed the antioxidant and spasmolytic activities of the infusion prepared of lemon verbena [6,7]. Verbena aqueous extract given to rats has proven the hypnotic effect of the plant by promoting sleep [8]. Polyphenols extracted from lemon verbena reduced the obesity burden and restored the mitochondrial activity through AMPK-dependent pathways [9]. 

Verbascoside (Vs), a major phenypropanoid glycoside, is the most abundant polyphenol in lemon verbena tea and its yield is reported to be around 3.94% (*w/w* dry weight of leaves) [10]. Vs contained in *Buddlejia davidii* and *Lippia multiflora* has already been proven to possess an antioxidant activity [11,12]. Vs has also shown an anti-inflammatory effect *in vitro* on macrophages and THP-1 cells [13,14]. Furthermore, Vs has been reported to exert an antimicrobial activity against *Staphylococcus aureus* and a neuroprotective effect, in vitro, on 1-methyl-4-phenylpyridinum ion-induced toxicity using PC12 cells [15,16]. Interestingly, intraperitoneal administration of Vs and lemon verbena aqueous and ethanolic extracts to mice promoted sleep and induced muscle relaxation, alongside alleviation of anxiety [17]. In addition to Vs, hastatoside (Hs) and verbenalin (Vn) are two abundant iridoids in verbena extract and have been proved to possess sleep-promoting effect [18]. To date, very little is known about the molecular mechanism by which lemon verbena or its compounds induce relaxation and act as anti-anxiety remedies.

In the present study, we investigated the effect of lemon verbena and Vs in mice and elucidated the molecular mechanisms underlying their effects in brain. Interestingly, the transcriptomic analysis in vivo showed regulation of genes implicated in activation of the mitochondrial function. Therefore, to confirm this finding we evaluated, in vitro, the effect of VEE and Vs on cells’ ATP production using SH-SY5Y, a Human neurotypic cell line. Also, we assessed the toxicity of VEE, Vs, Hs, and Vn, in addition to neuroprotective effect on dexamethasone (Dex) neurotoxicity.

## 2. Results

### 2.1. Effect of VEE and Its Compounds on SH-SY5Y Cells’Viability

**Figure 1:**
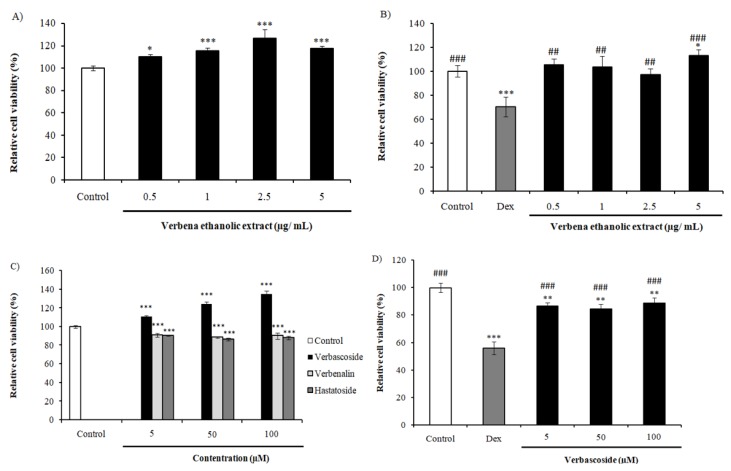
Relative cell viability of SH-SY5Y cells (**A**) treated with *Lippiacitriodora* ethanolic extract (VEE) at doses of 0.5, 1, 2.5 and 5 µg/mL, (**B**) co-treated with VEE and dexamethasone(Dex) (50 µM), (**C**) treated with verbascoside(Vs), hastatoside(Hs), and verbenalin(Vn) (5, 50 and 100 µM) and (**D**) co-treated with Vs and Dex (50 µM). Results were expressed in mean of cell viability ± SD. * *P* < 0.05; ** *P* < 0.001; *** *P* < 0.0001 compared with negative control group. # *P* < 0.05; ## *P* < 0.001; ### *P* < 0.0001 compared to Dex-treated group.

We performed the MTT assay to assess the effect of VEE on cell viability. We treated the cells with different concentrations of the extract which were 0.5, 1, 2.5 and 5 µg/mL of VEE. As shown in Figure 1A, all VEE concentrations increased cell viability significantly in a dose-dependent manner, with a higher value of 126.68 ± 7.81% at 2.5 µg/mL. The chemical analysis of various Verbenaceae plants, including *Lippia citriodora* and *Verbena officinalis*, showed a high abundance in Vs, also called acteoside, which is a phenylpropanoid glycoside [19,20,21,22,23,24]. In our study, we evaluated the cell viability of SH-SY5Y cells treated with 5, 50 and 100 µM of Vs, Hs and Vn. The results in Figure 1C show an increase of viable cells in a dose-dependent manner attaining 134.8 ± 3.8% at 100 µM in case of Vs. On the other hand, Hs and Vn decreased the cell viability significantly (Figure 1C). From these results, we selected Vs to be evaluated for its neuroprotective and energy metabolism effects.

In order to evaluate the neuroprotective activity, we used dexamethasone (Dex) as neurotoxic agent. VEE treatment protected SH-SY5Y cells fromDex toxicity with higher increase at 5 µg/mL (42.82% cell viability) (Figure 1B). Interestingly, cells co-treated with Vs and Dex showed an enhancement of cell viability by more than 30% compared to Dex-treated cells (Figure 1D). These data indicate neuroprotective effect exerted by VEE and Vs.

### 2.2. Effect of VEE on the Immobility Time of Mice

**Figure 2:**
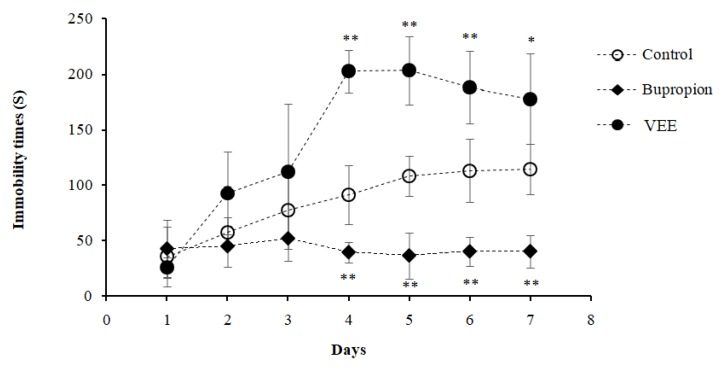
Effect of the oral administration of VEE (100 mg/Kg) and bupropion (20 mg/Kg) on mice immobility times in tail suspension test compared to the control (water 10 mL/Kg, p.o.). Results were expressed in mean of immobility time ± SD. * *P* < 0.05; ** *P* < 0.001 compared with Control group.

The tail suspension test (TST) was used to assess the antidepressant-like effect of VEE 100 mg/Kg compared to the control groups. Normally, drugs having an antidepressant effect decrease the immobility time of mice. In the present study, bupropion was used as a positive control, known for its antidepressant property. Bupropion-treated mice showed a decrease of immobility time on the 4th day of TST to 39.37 s compared to the initial test performed on the 1st day with a value of 42.52 s, resulting of the drug’s effect (Figure 2). As for the negative control group, the mice were fed with Milli-Q water and showed a gradual increase of immobility time to day 7 with 114.4 s compared to the initial test with a time of 35.48 s, proving an induction of depression on mice by TST, leading the animals to lack the desire to rectify themselves (Figure 2).

Interestingly, 100 mg/Kg body weight VEE-treated mice showed a highly significant increase of immobility time compared to negative and positive controls starting from day 4 of the test with 202.64 s, which gradually decreased to attain 177.63 s on the 7th day (Figure 2).The low immobility time of the depressant mice receiving only water compared the VEE-treated mice suggested that the effect observed was not a result of the stress induced by TST, but because of the induction of relaxation by VEE, which is a unique effect of VEE.

### 2.3. Elucidation of the Genes Regulated by VEE Treatment

To determine the molecular mechanism underlying the effect of VEE on immobility time, we analyzed the mice brains using DNA microarray to detect the transcriptomic changes. The analysis of the data revealed the up-regulation of 62 genes with a fold-change higher than 1.2, while 256 others were down-regulated below 0.65 fold-change. After annotating the genes, they were clustered in order to study their interactions and the pathways they are implicated in. Bupropion and VEE affected interesting pathways controlling the neuronal proliferation, spatial learning and memory, long-term potentiation and depression, inflammation and reactive oxygen species (ROS) production (Table 1). Interestingly, VEE treatment regulated genes such as *Adenylate cyclase (Ac)* implicated in the production of cyclic-Adenosine monophosphate (cAMP). It up-regulated the expression of genes implicated in calcium signaling including *Inositol 1,4,5-trisphosphate receptor type 2 (Itpr2)*, *Protein kinase C (Pkc)* and *Calcium channel voltage-dependent L type alpha 1C subunit (Cacna1c)* [25,26]. VEE treatment increased the expression of *Calcium/calmodulin dependent protein kinase IV (CamkIV)*, one of the genes stimulating mitochondrial biogenesis [27]. The expression of *cGMP-dependent protein kinase (Prkg1)* was affected by verbena treatment, which results in the induction of muscle relaxation [28]. Also, *5 hydroxytryptamine (serotonin) receptor 4 (Htr4)* involved in neurotransmitters production was enhanced, alongside with *AdenosineA2a receptor (Adora2)*, responsible of the development of several neurodegenerative diseases [29,30,31]. VEE enhanced the expression of *Dopamine receptor D1 (Drd1)*, implicated in activation of *Ac* [32]. 

As shown in Table 1, out of the all sets of genes, three were highly expressed in the case of VEE-treated mice, which are *Gelsolin (Gsn)*, *Transthyretin (Ttr)* and *Calcium/calmodulin-dependent protein kinase 2 inhibitor 1 (Camk2n1)*. Their expressions were increased 5.26, 3.72 and 2.19 fold, respectively. Recent studies showed a positive correlation between mitochondrial activity and expression of *Ttr* and *Gsn* [33,34]. As for *Camk2n1*, it has been shown to possess a role in controlling cell proliferation [35].

VEE treatment decreased the expression of *melanin-concentrating hormone receptor 1 (Mchr1)* to a fold-change equal to 0.55, while bupropion did not affect its transcription level. The down-regulation of this gene was found to enhance the metabolism [36], which implicates an activation of mitochondria. Also, *Mchr1* antagonist exerted an anti-depressant effect [37].

The *pro-melatonin-concentrating hormone (Pmch)* was drastically down-regulated (Table 1). It has been previously shown to exert a role in energy metabolism [38].

### 2.4. Validation of Expressions of Gsn, Ttr, Camk2n1 and Itpr2

**Figure 3:**
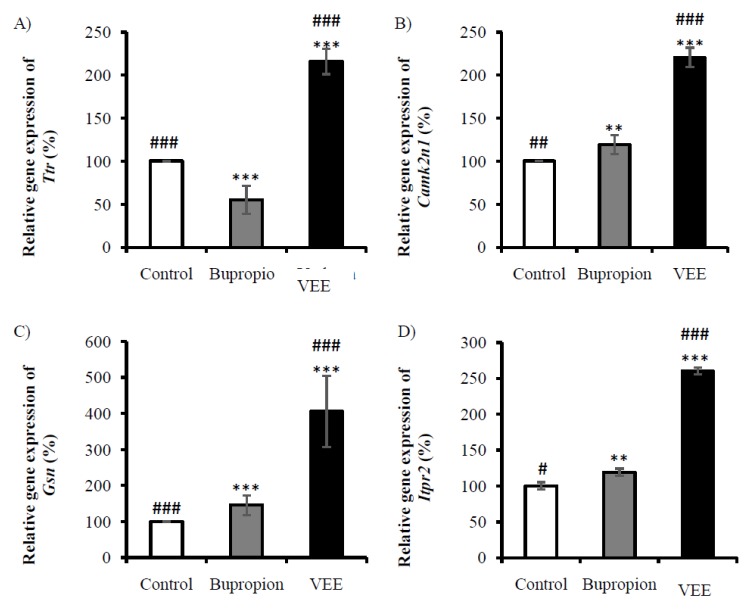
Validation of the expression of genes regulated by VEE treatment (100 mg/Kg) which are (**A**) *Ttr*, (**B**) *Camk2n1*, (**C**) *Gsn*, and (**D**) *Itpr2*. Results were expressed in relative gene expression ± SD. * *P* < 0.05; ** *P* < 0.001; *** *P* < 0.0001 compared with negative control group. # *P* < 0.05; ## *P* < 0.001; ### *P* < 0.0001 compared to bupropion-treated group.

The microarray analysis of brains collected from mice treated with 100 mg/Kg of VEE showed up-regulation of genes implicated in mitochondrial activity, with fold-changes higher than 2. These genes are *Gsn*, *Ttr*, and *Camk2n1*. Their up-regulations were confirmed and represented in relative gene expression, with the negative control expression as reference. Expressions of *Gsn*, *Ttr*, and *Camk2n1* were increased in the case of VEE-treated mice by 305% (relative gene expression), 115% and 110%, respectively (Figure 3A–C). The *Camk2n1* is an inhibitor that alters the transportation of Ca^2+^, responsible of the control of the intracellular amount of this ion to avoid its side effects.

*Itpr2* is responsible of intracellular calcium release. This gene was up-regulated by VEE treatment. Its expression was confirmed and showed an enhancement of 160% in VEE-treated mice compared to the control group. The effect of bupropion was not significant compared to VEE, with an increase of 19% (Figure 3D).

### 2.5. Antidepressant Effect of Low Doses of VEE and Vs

**Figure 4:**
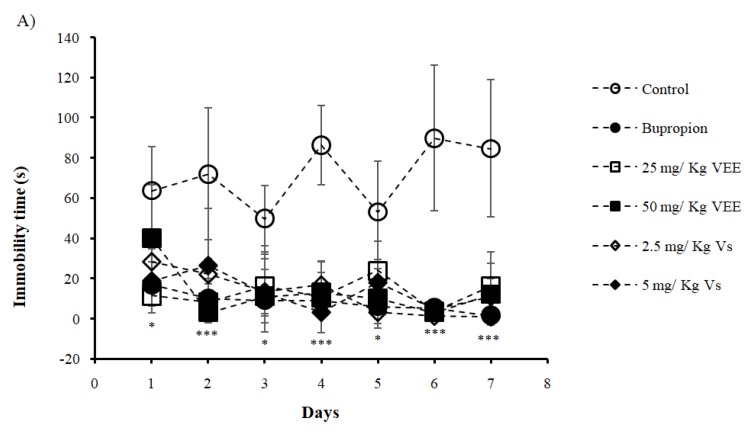

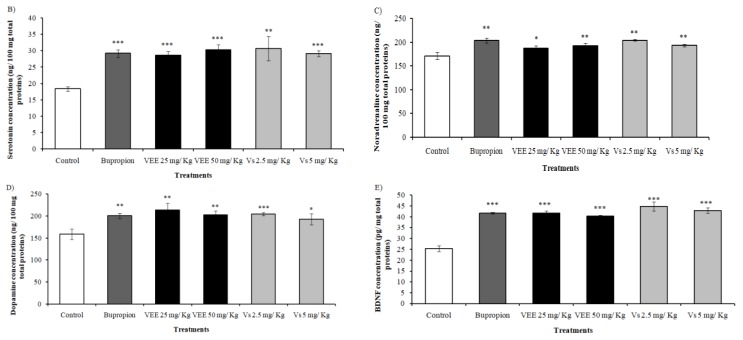
Effect of the oral administration of VEE (25 and 50 mg/Kg), Vs (2.5 and 5 mg/Kg) and bupropion (20 mg/Kg) on (**A**) mice immobility times in tail suspension test compared to the control (water 10 mL/Kg, p.o.) and their respective expression levels of (**B**) serotonin, (**C**) noradrenaline (**D**) dopamine and (**E**) BDNF. Results were expressed in mean of immobility time (s) and protein level ± SD. * *P* < 0.05; ** *P* < 0.001 and *** *P* < 0.0001 compared with Control group.

The control group showed higher immobility time compared to other treatments for 7 days of testing (Figure 4A). The immobility recorded on the first day was 63.81 s for the control, which increased to reach 84.33s on day 7. This increase proved induction of depression in mice. Bupropion treated mice scored an immobility time of 16.96 s on the first day and decreased to 1.56 s on the last day of the test, proving the antidepressant effect of bupropion. Results obtained on first day showed a significant difference between the control group and Vs and VEE at a dose of 25 mg/Kg. On the second day, VEE and Vs treatments decreased the immobility time and the scores were statistically comparable to the bupropion treated group, while the difference was highly significant compared to the control. Similar results were observed for the rest of the test, except on day 3 and 5 where the difference was not significant between the control group and the 25 mg/Kg VEE treated animals.

For decades, depression has been associated with levels of monoamines and catecholamines in the system [46]. Depressive patients have been found to present Sert and NA (norepinephrine) deficiency [47,48]. To confirm the antidepressant effect of the treatments on mice we quantified the amounts of Sert and NA in mice brains. The results showed a low concentration of Sert and NA for control group with an amount of 18 and 171 ng/100 mg total proteins, respectively (Figure 4B,C). Bupropion increased significantly Sert level by 61% compared to control group. A similar effect was observed in case of mice treated with VEE 25 and 50 mg/Kg and Vs 2.5 and 5 mg/Kg showing improvement of 57.90%, 67.05%, 69.19%and 61.04% total proteins, respectively. An enhancement of 19% was observed in NA level in case of bupropion treated mice. Also, the other treatments increased NA concentration with a higher rate of 19.35% for Vs 2.5 mg/Kg treated group.

One of the important targets of antidepressants is the dopaminergic system. We evaluated the effect of our treatments on dopamine levels in mice brains. Bupropion showed an increase of dopamine content by 26% (Figure 4D). The highest dopamine concentration, with an increase of 34.45%, was observed in mice treated with 25 mg/Kg of VEE. The lowest dopamine enhancement (21.21%) was obtained for mice treated with 5 mg/Kg of Vs.

Furthermore, we evaluated the concentration of BDNF, which is one of the markers of depression. Our findings showed an increase of BDNF levels in all treatments. Bupropion enhanced BDNF expression by 64.34% (Figure 4E). Interestingly, VEE at 25 mg/Kg and Vs at 2.5 and 5 mg/Kg were found to exert more substantial effect regarding BDNF level with an enhancement of 64.67%, 76.36% and 69.26%.

### 2.6. Evaluation of the Mitochondrial Activity of Cells Treated with VEE and Vs

**Figure 5:**
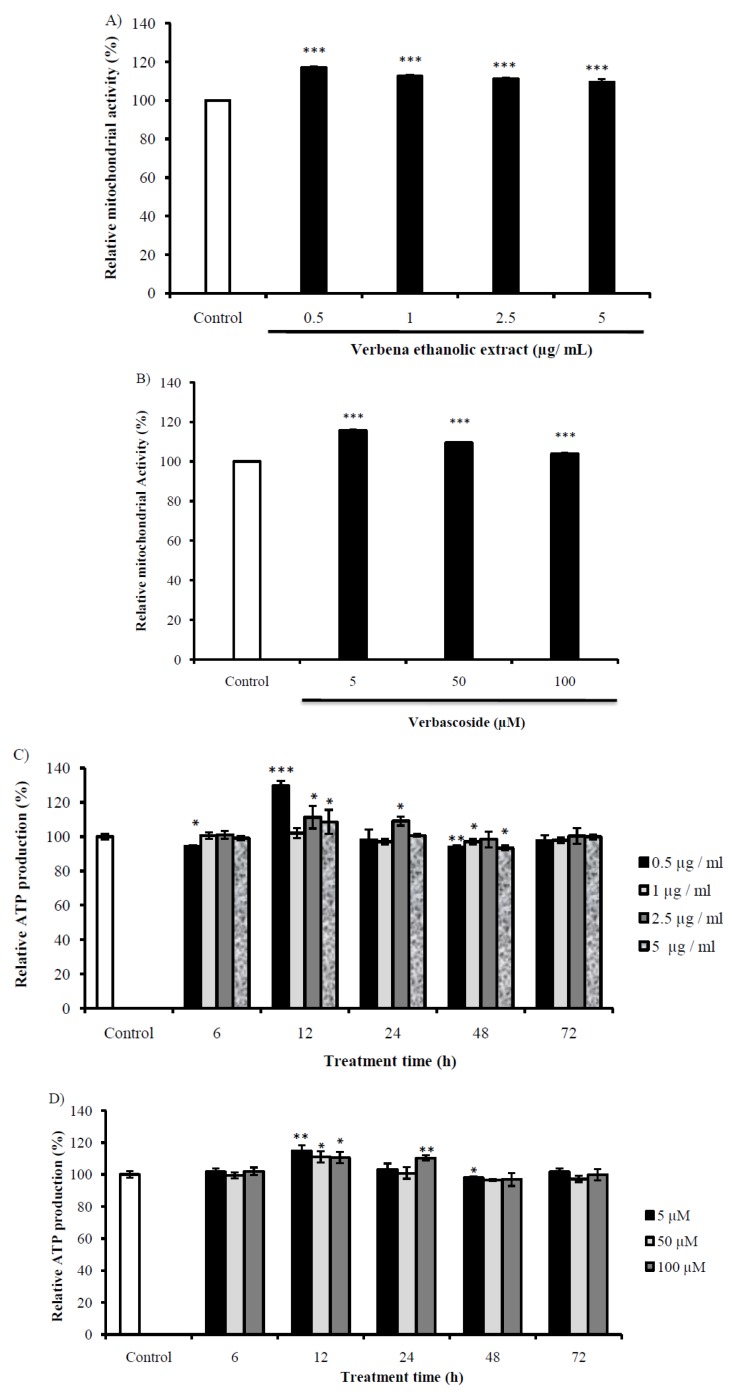
Evaluation of mitochondrial activity of SH-SY5Y cells treated with different concentrations of (**A**) VEE and (**B**) Vs. The intracellular ATP production of SH-SY5Y was assessed *in vitro* using the same concentrations of (**C**) VEE and (**D**) Vs at 6, 12, 24, 48 and 72 h. Results were expressed in mean of relative mitochondrial activity or ATP production (%) ± SD. * *P* < 0.05; ** *P* < 0.001; *** *P* < 0.0001 compared with control cells treated with Opti-MEM.

In order to measure the mitochondrial activity, we used the rhodamine 123 that stains the active mitochondria specifically. Both VEE and Vs induced mitochondrial activation of SH-SY5Y cells in a dose-dependent manner, with higher effect at lower concentrations. VEE at 0.5 µg/mL increased mitochondrial activity by 17% and its effect decreased to reach 9.37% for cells treated by 5 µg/mL (Figure 5A). Mitochondrial activity of cells treated with 5 µM of Vs was 115% compared to control, while the higher concentration enhanced the function only by 3% (Figure 5B). These results implicated a stimulation of energy production of VEE and Vs treatments.

The same concentrations of VEE and Vs were evaluated for their effect on energy generation by quantifying ATP level. As Figure 5C shows, VEE treatments were not effective on energy metabolism at 6 h, but they show a highly significant increase after 12 h, with a maximum of 129.71 ± 2.73%. The ATP content decreased in a time and dose-dependent manner to reach energy homeostasis after 72 h. Treating the cells with Vs increased ATP production significantly after 12 h (Figure 5D), which decreased gradually to attain the normal status at 72 h.These results proved the stimulation mitochondria by VEE and Vs.

### 2.7. Effect of VEE and Vs on Intracellular Calcium Levels

**Figure 6:**
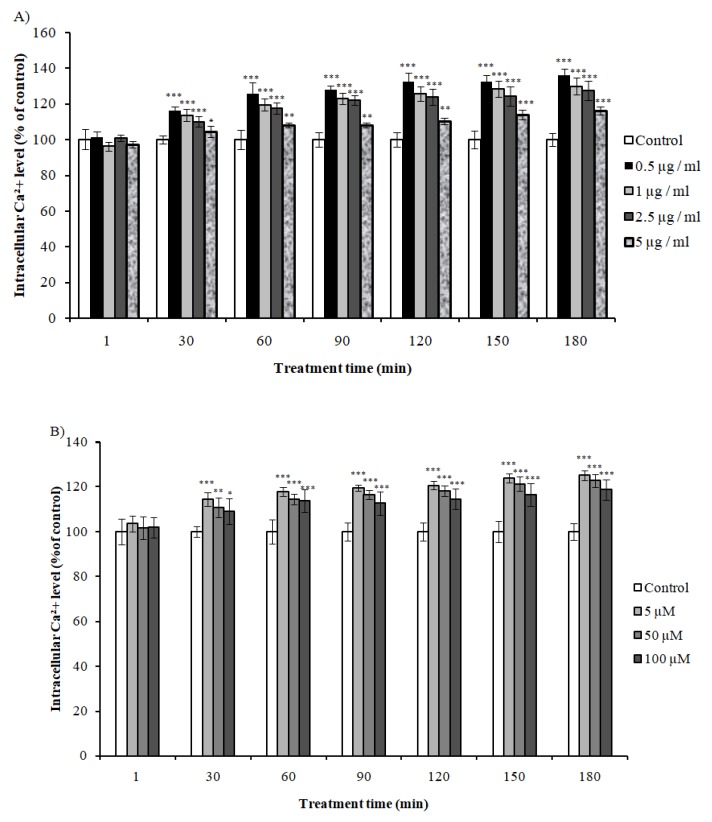
Evaluation of intracellular calcium levels of SH-SY5Y cells treated with different concentrations of (**A**) VEE and (**B**) Vs for 1–180 min. Results were expressed as percentage of control cells treated with Opti-MEM ± SD. * *P* < 0.05; ** *P* < 0.001; *** *P* < 0.0001 compared with control cells.

Studies have shown a correlation between intracellular calcium uptake and mitochondrial activation. Transcriptomic analysis showed regulation of genes involved in Ca^2+^ in cases of mice treated with VEE. Here, we evaluated the effect of VEE and Vs on Ca^2+^ levels on SH-SY5Y. VEE increased Ca^2+^ uptake after 30 min of treatment in concentration and time-dependent manner, with higher effect at lower concentrations (Figure 6A). Accordingly, Vs showed similar effect on Ca^2+^ with higher activity at lower doses (Figure 6B). These results proved the implication of Ca^2+^ in the observed activities, with Vs being responsible for VEE effects.

## 3. Discussion

Lemon verbena is a medicinal plant exerting important biological activities such as antidepressant, antioxidant, sleep-promoting and analgesic effects [24,49,50,51]. The molecular mechanisms underlying these effects are still unknown.

The *in vitro* study showed an increase of cell viability of VEE-treated cells compared to the non-treated cells, indicating an activation of cellular functionalities. Co-treatment of VEE and Dex enhanced the cell viability significantly compared to the Dex-treated cells. To determine the compound responsible for the effect observed, we treated the cells with the three most abundant compounds in the extract, Vs, Hs and Vn. The viability was enhanced by Vs in comparison to the control, while Hs and Vn were significantly decreased. The effect observed in the case of the extract is probably due to Vs. Also, Vs was tested for its neuroprotective effect and was found to alleviate Dex toxicity by more than 30%. These findings suggest that VEE and Vs have neuroprotective effects.

In the present work, we studied the effect of VEE on mice at the molecular level by analyzing the expression of all genes. We used the TST to induce psychological stress in mice. The TST results showed increase of immobility time of VEE-treated mice compared to both control groups. In 2017, Razavi et al. reported the anti-anxiety and muscle relaxant effects of VEE and Vs *in vivo* [17]. Another study showed induction of relaxation in mice and rats treated with essential oil extracted from the aerial part of verbena [50]. Accordingly, the aqueous extract of this plant was found to have a sedative effect in rats at high doses (700 and 1000 mg/Kg body weight of extract) [8]. Then, the increase of immobility time observed in this study may suggest the relaxant and sedative effects of VEE.

The evaluation of the transcriptome in the collected brains showed an enhancement of expression of genes implicated in the production of cAMP in the case of mice treated with VEE. *Drd1* expression was increased by VEE, while it remained stable in case of bupropion-treated mice. Previously, the enzymatic activity of *Ac* was found to be tightly regulated by *Drd1* through *Gβα* [32]. Also, VEE increased the expression of *Ac* in mice brains, the enzyme that was down-regulated by Bupropion treatment. Over-expression and activation of *Ac* by VEE implies an increase of cAMP generation, which has been associated with the induction of relaxation effect [52]. Accordingly, the use of apomorphine, a *Drd1* agonist, was proved to induce relaxation [53]. Moreover, treatment with the plant extract increased *Prkg1* expression, a gene that has been associated with induction of relaxation [28].

VEE affected the expression of genes modulating calcium homeostasis. *Itpr2* is one of the intracellular Ca^2+^ release channels, located in the membranes of endoplasmic and sarcoplasmic reticula. These are organelles are rich in Ca^2+^ion [25]. VEE up-regulated the expression of *Itpr2*, implicating an elevation of the ion in the cytosolic compartment. Ca^2+^-cytosolic content depends also on channels facilitating the transport of ion from the extracellular compartment [54]. One of these channels is *Cacna1c* which has been over expressed in VEE-treated mice. A previous study evaluated the transcriptomic changes induced by relaxation in humans and *Cacna1c* was found to by over-expressed [54]. Ca^2+^-induced increase by VEE, up-regulated the expression of *Pkc*, an enzyme found to be dependent to Ca^2+^ concentration in cells, and which activates *Ac* inducing an over-production of cAMP [41,55]. On the other hand, calcium homeostasis has been already proved to play an important role in muscle movement and walking behavior in humans. At the brain level, the calcium signaling regulates different functions, including signal transmission and also the learning and memory [56,57,58,59,60]. When accumulated in cytoplasm, the calcium is transported into mitochondria inducing the activation of enzymes implicated in generation of ATP, including ATP synthase and NADH^+^ dehydrogenase [61]. The inhibition of the calcium uptake by the mitochondria was found to increase the time needed for relaxation [62]. Accordingly, an increase of Ca^2+^ content has been proved to induce ATP production through cAMP generation [63]. These results suggest that VEE has a relaxant effect on mice through the generation of cAMP, which in addition to high intracellular Ca^2+^levels, induces activation of mitochondria.

For VEE-treated mice, *Gsn*, *Ttr*, and *Camk2n1* showed the highest expression levels compared to the set of genes analyzed by microarray, and the increase was more than 2 fold-changes, while mice receiving Bupropion showed a decrease of *Ttr* expression, whereas *Gsn* and *Camk2n1* expressions were slightly increased (less than 1.5 fold-change). Mutant mice over-expressing *Gsn* revealed an enhancement of respiratory chain activity [33]. Several studies have demonstrated the neuroprotective role of *Ttr* [64,65,66,67,68], and its positive correlation to mitochondrial function [34]. These findings proved an increase of mitochondrial activity, implying an over-production of ATP. VEE-treated group presented high level of *Camk2n1* expression compared to control group, which implicates a controlled cell proliferation. Previously, a study demonstrated the tumor suppressive effect of *Camk2n1* [35].

*Pmch* and *Mchr1* were significantly down-regulated by VEE. *Pmch*-deficient mice, as well as *Mchr1*-deficient mice, were found to be more active than wild type mice, and showing an increase in metabolic rate [36,38]. A specific *Mchr1* antagonist has showed antidepressant and anxiolytic effect [37]. The increase of immobility time of VEE-treated mice is due to the relaxant effect of the plant extract, and the molecular analysis proved its antidepressant effect.

In order to evaluate the effect of lower doses of VEE and their respective Vs contents, a second TST was conducted. The treatments used were 25 and 50 mg/Kg of VEE and 2.5 and 5 mg/Kg of Vs. Interestingly, the results showed a decrease in immobility time compared to the control group, and scores were statistically comparable to bupropion treated mice. Our findings suggest low doses have an antidepressant effect. In accordance with the transcriptomic analysis conducted here above, VEE and Vs might be induced mitochondrial activation through accumulation of cAMP and Ca^2+^, to a lesser extent than high dose of VEE, resulting in agitation of mice rather than their relaxation. To prove the antidepressant effect observed in vivo, we evaluated the levels of different depression markers. Sert and NA implication in depression has been documented and are considered as targets of antidepressants [47,48]. In our study, VEE and Vs were found to enhance Sert and NA levels demonstrating an antidepressant effect of the treatments on mice.

Previous studies found that antidepressants targeting the expressions of Sert and NA only present limitations. Patients might show movement delay, lack of concentration or even persistence of anhedonia [69]. Accordingly, drugs acting on the dopaminergic system have been developed. Dopamine is a catecholamine responsible of expression of emotions such as pleasure and motivation, and stimulates concentration [69]. Hence, we assessed dopamine levels in brains. The results showed a highly significant increase of dopamine expression by VEE and Vs compared to control group. These findings prove the antidepressant activity of VEE and Vs by stimulating the pleasure mechanism.

It has been documented that antidepressants acting on serotonergic and norepinephric mechanisms lead to enhancement of BDNF levels in rodents [70,71]. We evaluated the effect of treatments on BDNF in brains. The results showed a highly significant increase of BDNF by VEE and Vs treatments. Also, it has been documented that Ca^2+^ and cAMP levels regulate BDNF expression through CREB (cAMP response element-binding protein) [72]. 

The results obtained *in vivo* revealed the activation of mechanisms responsible for the increase of cytosolic Ca^2+^ and cAMP generation, messengers inducing the mitochondrial activity. To confirm this hypothesis, we evaluated the effect of VEE on mitochondrial activity. The results showed enhancement of mitochondrial function in a concentration-dependent manner. Accordingly, Vs increased mitochondrial function in a similar tendency as VEE.ATP production *in vitro* was evaluated to confirm the effect of VEE and Vs mitochondrial activity. Human neurotypicSH-SY5Y cells treated with VEE showed a significant increase of ATP content in a dose-dependent manner after12h treatment. Energy metabolism gradually decreased to regain the initial state. Vs is one of the most important compounds contained in VEE, and has been proven to induce muscle relaxation in mice [17]. Next, we evaluated the potential effect of Vs on mitochondrial activity. We observed that Vs-treatment also showed an increase in ATP production at 12h, which restored to its original condition progressively. In 2013, Bhasin et al. evaluated the transcriptomic changes in humans in response to relaxation condition and showed regulation of genes activating energy metabolism [54]. ATP increase has been found to be regulated positively by activation of mitochondrial calcium uptake, as aresult of different stimuli such as alimentation, hormones and neurotransmitters [61,73,74,75,76]. Our *in vitro* study showed that VEE and Vs enhanced intracellular calcium levels in a concentration and time-dependent manner with similar tendency as mitochondrial activation. These results proved the increase of calcium and energy metabolism related genes regulated by the treatments *in vivo*.

## 4. Materials and Methods 

### 4.1. Plant Material and Extraction Method

The leaves of *Lippia citriodora* were collected in July 2016 from Marrakech Region (Morocco). The species was authenticated by Prof. Ahmed Ouhammou from Cadi Ayyad University, Faculty of Sciences Semlalia, Department of Biology, Marrakech, Morocco. A voucher specimen of plant material (MARK-11186) was deposited in the Herbarium of the same institution. After air drying, the plant material was crushed by a mortar and extracted with ethanol 70%, with a ratio plant material/solvent of 10% (*w/v*). The extraction was carried out in the dark for 2 weeks and vigorously shacked twice a day. The extract was centrifuged and the supernatant filtered through 0.22 µm Millipore (Mark Millipore, Carrigtwohill, Ireland) and solvent evaporated by a rotary evaporator. The yield of VEE was 13.3%.

### 4.2. Chemicals

Vs, Hs and Vn were purchased from Sigma Aldrich, USA. Dulbecco’s Modified Eagle Medium (DMEM)/F-12 and Opti-MEM were obtained from Gibco, USA. Fetal bovine serum was from Gibco, South America. Penicillin - Streptomycin were purchased from Biowhittaker, USA. Non-essential amino acids were from Cosmo Bio Co, LTD, Japan. MTT (3-(4,5-dimethylthiazol-2-yl)-2,5-diphenyltetrazolium bromide) and dexamethasone were from Dojindo, Japan. Bupropion was from Wako, Japan. ATP bioluminescence kit was from TOYO Ink, Japan. ISOGEN kit was purchased from Nippon Gene, Japan. RIPA lysis buffer was from (Santa Cruz Biotechnology, USA). 2-D Quant was purchased from GE Healthcare Life Sciences, USA. Calcium Kit II-Fluo 4 was from Dojindo, Japan.

### 4.3. Cell Culture

The *in vitro* experiments were conducted on SH-SY5Y cells. This neurotypic cell line was obtained from America Type Culture Collection, Manassas, USA. Cells were maintained in Dulbecco’s Modified Eagle Medium (DMEM)/F-12, supplemented with 15% fetal bovine serum, 1% Penicillin (5000 µg/mL) Streptomycin (5000 IU/mL) and 1% of non-essential amino acids. The culture was incubated at 37 °C in a humidified atmosphere of 5% CO_2_ incubator. Opti-MEM, a reduced serum medium, was used to culture cells for the evaluation of cell viability and intracellular ATP.

### 4.4. Determination of Cell Viability

3-(4,5-dimethylthiazol-2-yl)-2,5-diphenyltetrazolium bromide (MTT) assay was used to assess cell viability of SH-SY5Y cells. Briefly, the cells were seeded in a 96-well plate (fibronectin-coated plate) (BD BioCoat, New York City, NY, USA) with a density of 2 × 10^5^ cell/mL. After 24 h, the medium was removed and the cells were VEE (0.5, 1, 2.5 and 5 µg/mL), Vs, Hs or Vn (3.1, 31.2 and 62.4 µg/mL) diluted in Opti-MEM. After 72h incubation period, 10 µL MTT (5 mg/mL) mixed with 100 µL of Opti-MEM was added to each well and the plate was incubated for further 6 h. The formazan crystals formed by the mitochondrial activity were solubilized by adding 100 µL of 10 % SDS (*w/v*). The absorbance was measured at 570 nm using a microtiter plate reader (Dainippon Sumitomo Pharma Co., Ltd., Tokyo, Japan). The results were expressed in percentage of relative cell viability.

To evaluate the neuroprotective effect of VEE, Vs, Hs and Vn, the cells were co-treated with DEX (50 µM), incubated for 72h, then their viability assessed by MTT assay as described above.

### 4.5. Animals

Male ICR mice, 3 weeks old, weighting between 20 and 30 g were purchased from Charles River laboratories (Tokyo, Japan). Mice were housed individually and had access to food and water *ad libitum*, in a controlled environment (56% humidity, 23 °C temperature, 12/12 h light/dark cycle). Before starting the oral administration and the tail suspension test, the mice were allowed to acclimatize for one week. All experiments were performed in strict accordance with NIH guidelines and were approved by the Animal Ethics Committee of the University of Tsukuba, Japan. The ethical approval code is 16-042 (1/06/2016).

### 4.6. Tail Suspension Test

The animals were divided into three groups. A negative control group receiving Milli-Q water (10 mL/Kg; *n* = 6), a positive control group treated with 20 mg/Kg of Bupropion (*n* = 7) and VEE-treated group (*n* = 7) which received a dose of 100 mg/Kg. The samples were administrated orally every day for 7 days.

The tail suspension test (TST) is a widely used technique to screen the antidepressant effects of drugs. The methodology used in this study is as described by Steru et al., 1985 [77]. Briefly, the TST was performed 60 min after the administration of treatments. The duration of the test was 6 min and the immobility time was measured on the last 4 min of the test. A mouse was considered immobile only when it is hanged passively, showing no resistance to the stress applied by the test. The experiment was recorded using a camera and scored by observing the videos. After completion of the behavioral test, mice were sacrificed by cerebrospinal dislocation, then the whole brains were collected for the subsequent analysis.

A second TST was conducted to evaluate the effect of lower doses of VEE (25 mg/Kg, *n* = 6; 50 mg/Kg, *n* = 7) and their respective Vs content (2.5 mg/Kg, *n* = 6; 5 mg/Kg body weight, *n* = 7) on mice. HPLC analysis showed that VEE contains 10% of Vs (data not shown). Other control groups (Milli-Q, *n* = 6; Bupropion 20 mg/mL, *n* = 7) were used for the second test. TST was performed according to the protocol previously described.

### 4.7. DNA Microarray Analysis

The total RNA was extracted from the brain tissues previously collected using ISOGEN kit and quantified by Nanodrop 2000 spectrophotometer (Thermo Fisher scientific, Wilmington, NC, USA). 

To elucidate the molecular mechanism underlying the effect of VEE on neuronal activities, we evaluated the total gene expression of brain tissues by performing microarray on RNAs previously extracted. The experiment was conducted according to the Affymetrix Genechip 3’ IVT PLUS reagent kit user’s guide. Briefly, the RNAs were reverse transcripted to generate double stranded DNA. The latter used as a template to synthesize the Biotin-labeled cRNA. After fragmentation of the labeled cRNA, the mixture was hybridized to the Affymetrix mouse 430 PM array strips (Affymetrix) for 16 h at 45 °C in the hybridization station. In Geneatlas Fluidics station, the hybridized arrays were washed and stained, then scanned using Geneatlas imaging station. The total number of genes analyzed by this method is 39,396 genes. All brain samples were analyzed by microarray. The data obtained were analyzed by Expression Console and Pathway Studio software and DAVID and Consensus Path databases.

### 4.8. Real Time Polymerase Chain Reaction (qRT-PCR)

RNA extracts obtained from mice brains were used as templates to validate the microarray results through evaluation of the expression level of some relevant genes regulated by Verbena treatment. First, a reverse transcription was performed, using the Superscript IV reverse transcriptase kit (Invitrogen, Carlsbad, CA, USA) following the manufacturer’s protocol. Briefly, we incubated a mixture of RNA samples (0.2 µg/µL) and Oligo(dT)_12-18_/dNTP (0.5 µg/µL; 10 mM) for 5 min at 65 °C, and then placed for 1 min on ice. The Reverse transcriptase solution was added and incubated the samples at 42 °C for 60 min and then 10 min at 60 °C. The cDNA produced is used to evaluate the expression of 3 genes: *Gelsolin “Gsn”* (Mm00456679_m1), *Transthyretin “Ttr”* (Mm00443267_m1), *Calcium/calmodulin-dependent protein kinase II inhibitor 1 “Camk2n1”* (Mm01718432_s1) and *Inositol 1,4,5-trisphosphate receptor type 2 “Itpr2”* (Mm00444937_m1). This experiment was conducted using TaqMan Universal PCR mix and TaqMan Probes and the amplifications were performed in a 7500 Fast Real-time PCR (Applied Biosystems, Foster City, CA, USA) with the following conditions: 50 °C for 2 min, followed by 95 °C for 10 min, and 40 cycles of 95 °C for 15 s followed by 60 °C for 1 min.

### 4.9. Quantification of Neurotransmitters and BDNF

To confirm the antidepressant effect of VEE and Vs at lower doses, we quantified the levels of serotonin (Sert), noradrenaline (NA), dopamine and BDNF in brains. The proteins were measured in the frontal cortex. First we homogenized 100 mg of tissue in 1 mL of RIPA buffer. The homogenate was centrifuged for 5 min at 10,000× *g* and 4 °C. The supernatant was collected and stored at −80 °C. The dopamine, Sert and NA were quantified using ELISA kits (Immusmol SAS, Talence, France). BDNF was measured by colorimetric sandwich ELISA kit (Proteintech, Rosemont, IL, USA). The experiments were conducted following the manufacturer’s instructions. The results of each treatment group were corrected by their respective total protein content determined using 2-D Quanti kit.

### 4.10. Measurement of Mitochondrial Activity

Mitochondrial function was measured using rhodamine 123, a fluorescent dye. The protocol was as described previously by Matsukawa et al., 2017 [78]. Briefly, treated SH-SY5Y were incubated for 20 min at 37 °C after addition of rhodamine 123 (10 µg/mL). Cells were lysed by 1% Triton X-100 and the fluorescence intensity of rhodamine (excitation/emission 485/528 nm) was measured.

### 4.11. Measurement of the Intracellular ATP Production

The mitochondrial activity was assessed by measuring the intracellular ATP content of cells using ATP bioluminescence kit. Cells were cultured (2 × 10^5^ cell/mL) in a 96-well plate (fibronectin-coated plate) and treated with different concentrations of VEE and Vs for 6, 12, 24, 48 and 72 h. The cells were lysed and the ATP content measured by adding 100 µL of luciferin-luciferase solution. The luminescence was measured using the microtiter plate reader (Dainippon Sumitomo Pharma Co., Ltd., Japan).

### 4.12. Measurement of Intracellular Calcium Level

Calcium Kit II-Fluo 4 was used to measure intracellular calcium levels of SH-SY5Y. The measurement was conducted according to the manufacturer’s protocol. Briefly, SH-SY5Y cells were seeded in black clear-bottom 96 well plates (Corning, NY, USA) and then treated with loading buffer (5% Pluronic F-127, 250-mmol/L Probenecid and 1-μg/μLFluo 4 AM in Hanks’–HEPES Buffer) for 1 h. The supernatant was removed and cells were washed with PBS. Cells were treated with VEE and Vs as described previously. Fluorescence intensity (excitation/emission 485/528 nm) was measured every 30 min using a Powerscan HT plate reader.

### 4.13. Statistical Analysis

Results are expressed as means ± SD, and statistical analyses were performed using a Student’s *t*-test using IBM SPSS Statistics 23 software. Differences were determined statistically significant at a *P*-value of less than 0.05.

## 5. Conclusions

Taken together, our findings suggest that, depending on the administered dose, VEE and Vs induce either relaxation or anti-depression effects. Higher doses of VEE induced relaxation through regulation of genes, including *Itpr2* and *Ac*, *responsible* for Ca^2+^ and cAMP generation. Lower doses of VEE and their respective Vs amount treatments was found to induce antidepressant-like effects by enhancing the BDNF, NA, Sert and dopamine expressions, which are cAMP and Ca^2+^ dependent. VEE and Vs increased Ca^2+^ intracellular levels leading to the enhancement of mitochondrial activity and ATP concentration. The effects of VEE observed *in vivo* and *in vitro* are due mostly to Vs.

## Figures and Tables

**Table 1:** Genes regulated by VEE involved in induction of relaxation and the activation of energy metabolism. The ratios were calculated using the data of mice receiving water as reference.

Gene ID	Gene Name	Verbena Ratio	Bupropion Ratio	Function
***Gsn***	*Gelsolin*	5.26	1.54	Amyloid beta peptides aggregation [33,39]
***Ttr***	*Transthyretin*	3.72	3.91	
***Camk2n1***	*Calcium/calmodulin-dependent protein kinase 2 inhibitor 1*	2.19	1.03	Tumor suppressor [35]
***CaMK4***	*calcium/calmodulin-dependent protein kinase IV*	1.46	1.20	Long-term memory [40]
***Cacna1c***	*Calcium channel, voltage-dependent, L type, alpha 1C subunit*	1.45	1.07	Cytosolic calcium content [26]
***Pkc***	*Protein kinase c*	1.45	0.98	*Adenylate cyclase* activation [32,41]
***Drd1***	*Dopamine receptor 1*	1.43	1.07	
***Adora2***	*Adenosine A2a receptor*	1.34	1.1	Cyclic-Adenosine monophosphate (cAMP) production [42]
***Htr4***	*5 hydroxytryptamine (serotonin) receptor 4*	1.34	1.25	Modulation of neurotransmitter release [29]
***Itpr2***	*Inositol 1,4,5-trisphosphate receptor type 2*	1.30	1.22	Intracellular calcium release [25]
***Ac***	*Adenylate cyclase*	1.28	0.85	Production of cAMP [43]
***Prkg1***	*cGMP-dependent protein kinase 1*	1.25	1.32	Induction of relaxation [28]
***Mchr1***	*melanin-concentrating hormone receptor*	0.55	1.01	Inhibition of cAMP accumulation [44]
***Pmch***	*pro-melanin-concentrating hormone*	0.12	0.12	Melanin-concentrating hormone activity [45]

## Abbreviations

Ac	Adenylate cyclase
Adora2	Adenosine A2a receptor
Cacna1c	Calcium channel, voltage-dependent, L type, alpha 1C subunit
Camk2n1	Calcium/calmodulin-dependent protein kinase II inhibitor 1
Camk4	Calcium/calmodulin-dependent protein kinase IV
Dex	Dexamethasone
Drd1	Dopamine receptor 1
Gsn	Gelsolin
Hs	Hastatoside
Htr4	5 hydroxytryptamine (serotonin) receptor 4
Itpr2	Inositol 1,4,5-trisphosphate receptor type 2
Mchr1	Melanin-concentrating hormone receptor
MTT	3-(4,5-dimethylthiazol-2-yl)-2,5-diphenyltetrazolium bromide
NA	Noradrenaline
Pkc	Protein kinase c
Pmch	Pro-melanin-concentrating hormone
Prkg1	cGMP-dependent protein kinase 1
Sert	Serotonin
TST	Tail suspension test
Ttr	Transthyretin
VEE	Verbena ethanolic extract
Vn	Verbenalin
Vs	Verbascoside
